# Supplementary material for: Tetramerisation of the CRISPR ring nuclease Crn3/Csx3 facilitates cyclic oligoadenylate cleavage
Source: eLife. 2020 Jun 29;9:e57627. doi: 10.7554/eLife.57627 (PMC7371418; doi:10.7554/eLife.57627)
Supplement: Supplementary file 1. [file elife-57627-supp1.docx]

**Supplementary Table 1. Data collection and refinement statistics for H60A mutant of Csx3 in complex with cA_4_.**

|  | Csx3 H60A mutant + cA_4_ |
| --- | --- |
| Data collection |  |
| Space group | C 1 2 1 |
| Cell dimensions |  |
| *a*, *b*, *c* (Å) | 194.0, 60.4, 107.1 |
| α, β, λ (°) | 90, 116.5, 90 |
| Resolution (Å) | 53.56 – 1.84 (1.87 – 1.84)* |
| *R*_sym_ or *R*_merge_ | 0.04 (0.72) |
| *I* / σ*I* | 11.3 (1.1) |
| Completeness (%) | 96.9 (70.0) |
| Redundancy | 3.2 (2.4) |
|  |  |
| Refinement |  |
| Resolution (Å) | 53.56 – 1.84 |
| No. reflections | 88795 |
| *R*_work_ / *R*_free_ | 0.20 / 0.24 |
| No. atoms | 8639 |
| Protein | 7621 |
| Ligand/ion | 578 |
| Water | 440 |
| *B*-factors |  |
| Protein | 37.0 |
| Ligand/ion | 43.4 |
| Water | 38.00 |
| R.M.S. deviations |  |
| Bond lengths (Å) | 0.016 |
| Bond angles (°) | 1.76 |

* Values in parentheses are for the high resolution shell.
