# Supplementary material for: Tetramerisation of the CRISPR ring nuclease Crn3/Csx3 facilitates cyclic oligoadenylate cleavage
Source: eLife. 2020 Jun 29;9:e57627. doi: 10.7554/eLife.57627 (PMC7371418; doi:10.7554/eLife.57627)
Supplement: Supplementary file 2. [file elife-57627-supp2.docx]

**Supplementary Table 2.** *Dynamic light scattering studies with AfCsx3.* In the absence of cA_4_, AfCsx3 (80 µM) forms particles with a molecular weight of ~36 ± 9 kDa consistent with protein in a homodimeric state. When an equimolar amount of cA_4_ is added, Csx3 forms multiple large molecular weight species (three species detected in each replicate) with significantly greater particles sizes (denoted by increase in the particle Z-average). The mass (%) per volume of each of these species is indicated alongside its molecular weight.

| Replicate | **AfCsx3** | | | **AfCsx3 + cA_4_** | | |
| --- | --- | --- | --- | --- | --- | --- |
|  | Est. MW (kDa) | Mass (%) | Z-Average (nm) | Est. MW (kDa) | Mass (%) | Z-Average (nm) |
| 1 | 36.3 ± 8.8 | 100 | 5.53 ± 1.33 | 453 ± 10.1 | 66.2 | 16.2 ± 0.361 |
|  |  |  |  | 561 ± 12.3 | 31.6 | 17.8 ± 0.368 |
|  |  |  |  | 438 ± 102 | 0.3 | 42.9 ± 0.667 |
| 2 | 37.7 ± 9.5 | 100 | 5.55 ± 1.44 | 237 ± 14.5 | 32.8 | 12.3 ± 0.722 |
|  |  |  |  | 504 ± 13.4 | 33.1 | 17.0 ± 0.348 |
|  |  |  |  | 10400 ± 706 | 29.5 | 62.0 ± 4.07 |
| 3 | 36.4 ± 9.0 | 100 | 5.51 ± 1.37 | 50.7 ± 2.60 | 33.2 | 6.45 ± 0.326 |
|  |  |  |  | 1810 ± 124 | 30 | 29.6 ± 1.60 |
|  |  |  |  | 4080 ± 333 | 32.4 | 54.8 ± 0.940 |
